# Gender norms and mass deworming program access in Comé, Benin: A qualitative assessment of gender-associated opportunities and challenges to achieving high mass drug administration coverage

**DOI:** 10.1371/journal.pntd.0008153

**Published:** 2020-04-17

**Authors:** Rachel E. Geyer, Moudachirou Ibikounlé, Mira Emmanuel-Fabula, Amy Roll, Euripide Avokpaho, Abiguel Elijan, Léopold Codjo Wèkè, Comlanvi Innocent Togbevi, Félicien Chabi, Parfait Houngbégnon, Adrian J. F. Luty, Elodie Yard, Judd L. Walson, Susan Graham, Arianna Rubin Means

**Affiliations:** 1 Department of Global Health, University of Washington, Seattle, United States of America; 2 Département de Zoologie, Faculté des Sciences et Techniques, Université d’Abomey-Calavi 01BP526, Cotonou, Benin; 3 Institute de Recherche Clinique du Bénin, Cotonou, Benin; 4 Université de Paris, MERIT, IRD, F-75006 Paris, France; 5 Division of Life Sciences, Natural History Museum, London, United Kingdom; 6 Department of Medicine, University of Washington, Seattle, United States of America; 7 Department of Epidemiology, University of Washington, Seattle, United States of America; World Health Organization, INDONESIA

## Abstract

The World Health Organization’s Neglected Tropical Disease Roadmap has accelerated progress towards eliminating select neglected tropical diseases (NTDs). This momentum has catalyzed research to determine the feasibility of interrupting transmission of soil-transmitted helminths (STH) using community-wide mass drug administration (MDA). This study aims to identify potential gender-specific facilitators and barriers to accessing and participating in community-wide STH MDA, with the goal of ensuring programs are equitable and maximize the probability of interrupting STH transmission. This research was conducted prior to the launch of community-wide MDA for STH in Comé, Benin. A total of 10 focus group discussions (FGDs) were conducted separately among 40 men, 38 women, and 15 community drug distributors (CDDs). Salient themes included: both men and women believe that community-wide MDA would reduce the financial burden associated with self-treatment, particularly for low income adults. Community members believe MDA should be packaged alongside water, sanitation, and other health services. Women feel past community-wide programs have been disorganized and are concerned these distributions will be similar. Women also expressed interest in increased engagement in the implementation of future community-based public health programs. Men often did not perceive themselves to be at great risk for STH infection and did not express a high demand for treatment. Finally, the barriers discussed by CDDs generally did not align with gender-specific concerns, but rather represented concerns shared by both genders. A door-to-door distribution strategy for STH MDA is preferred by women in this study, as this platform empowers women to participate as health decision makers for their family. In addition, involving women in planning and implementation of community-wide programs may help to increase treatment coverage and compliance.

## Author summary

Soil-transmitted helminths (STH) affect an estimated 1.5 billion people globally. The DeWorm3 Project is investigating if it is possible to interrupt transmission of STH with community-wide deworming of individuals of all ages. We conducted focus group discussions with adult men, women, and community drug distributors (CDDs) in Comé, Benin to identify gender-specific facilitators and barriers to accessing and participating in community-wide mass drug administration (MDA) programs. Our findings suggest that door-to-door community-wide MDA may not only improve MDA treatment coverage by increasing access but may also empower women by facilitating increased decision making on behalf of their regarding who is treated and where. Additionally, women may be an untapped resource for strategically reaching missed populations and women reported seeking increased involvement within the deworming of their families. Men did not perceive themselves to be at risk and thus did not have high demand for deworming. Both genders reported that they struggle to afford deworming drugs outside of MDA and that MDA coverage would be higher if treatment was integrated with water and sanitation services. Finally, CDDs generally identified only barriers and facilitators to MDA that were shared by both genders and may require more training to ensure MDA programs are equitable and effectively engage all at risk populations.

## Introduction

The World Health Organization (WHO) estimates that 1.5 billion people are infected with soil-transmitted helminths (STH) globally, including hookworm species (*Necator americanus* and *Ancylostoma duodenale*), roundworms (*Ascaris lumbricoides*), and whipworms (*Trichuris trichiura*) [1]. Heavy STH infections are associated with morbidities including iron-deficiency anemia, malnutrition, growth faltering, and cognitive deficiencies [2,3]. Current WHO guidelines focus specifically on the control of STH-associated morbidities, targeting empiric treatment to individuals who disproportionally experience them, including school and preschool-aged children and women of reproductive age [4,5]. However, while the current school-based standard-of-care is effective in reducing population-level morbidity due to STH, community-wide mass drug administration (MDA) of individuals of all ages may address persistent challenges associated with adult reservoirs of infection and associated onward STH transmission in the community [6].

The WHO Neglected Tropical Disease (NTD) Roadmap and London Declaration have accelerated progress toward eliminating selected NTDs, and formalized long-term disease-specific goals for other NTDs [7,8]. This political momentum has catalyzed research initiatives such as the DeWorm3 Project, which aims to determine the feasibility of interrupting STH transmission using a community-wide MDA platform [9]. The success of STH transmission interruption efforts is predicated on achieving both high levels of treatment coverage (the proportion of individuals offered treatment) and high levels of compliance (the proportion of targeted individuals effectively treated). Gender and other intersectional aspects of equity in healthcare access can influence treatment coverage and compliance and, in effect, may impact the probability of transmission interruption [10–13].

Understanding gender-specific barriers to access and participation in MDA is necessary to ensure that community-wide STH MDA programs are delivered equitably and with high treatment coverage. Previous studies around gender and NTD MDA programs found there is a need for community health programs to tailor themselves to the needs of both men and women participants [10–12]. In this qualitative study, we evaluated perceived facilitators and barriers to community-wide MDA delivery and how these perceptions differ between men and women in the DeWorm3 study site of Comé, Benin. We also examined if and how volunteer community drug distributor (CDD) perceptions of barriers and facilitators align with gender-specific concerns. In order for community-wide MDA programs to achieve high treatment coverage, CDDs will likely need to be aware of gender-specific barriers to MDA and tailor activities appropriately to ensure that all community members, including women, can make informed health decisions [10,11]. By addressing gender-specific barriers to participation, MDA campaigns can potentially reach and engage populations who are prone to being systematically missed by treatment. Simultaneously, it is important to consider that public health programs such as large-scale MDA campaigns inherently interact with the socio-cultural norms of a population, and thus through their very design have the potential to move the pendulum on how different populations are (or are not) accessing health programming [10]. In addition, gender intersects on other axes of inequality, such as education and income, which may play a role in how individuals participate in MDA campaigns. In this study we utilize a Gender Based Analysis (GBA) model to evaluate gender-specific opportunities and challenges to deliver community-wide MDA for STH with high coverage and, as a result, to identify potential pathways through which MDA programs may serve as mechanisms for women’s empowerment [14].

## Methods

### Ethics statement

The DeWorm3 Project in Benin, led by the Institut de Recherche Clinique du Bénin (IRCB) in Calavi, was reviewed and approved by the National Ethics Committee for Health Research (002-2017/CNERS-MS) from the Ministry of Health (MOH) in Benin. The study was also approved by The Human Subjects Division at the University of Washington (STUDY00000180). ClinicalTrials.gov Identifier: NCT03014167. All participants were adults 18 years of age or older. Participants provided written consent to participate in focus group discussions (FGDs). Or, if participants were unable to sign the consent forms they applied a thumbprint using an inkpad, and their name was written on the same page by an impartial witness of the participant’s choice who was also present for the consenting process.

This study is nested within the DeWorm3 Project, a community cluster randomized trial across three countries testing the feasibility of interrupting transmission of STH. The study design has been described in-depth elsewhere [9]. This study was conducted in the Commune of Comé, Benin prior to the first round of community-wide MDA, as part of the trial’s formative qualitative research. This formative research was conducted to inform a baseline impression of barriers and facilitators of deworming within these communities and to inform design and optimized delivery of subsequent rounds of MDA in the DeWorm3 Project.

### Study site

Comé, Benin previously had 5 years of school-based MDA targeting school-aged children. The lymphatic filariasis (LF) program was also active in Come until 2007, when the area transitioned to post-MDA surveillance status.

Benin has a population of roughly 11 million people, with 40% of households living below the poverty line [15,16]. All three STH species are endemic to Benin, with a national prevalence of 22.7% and up to 650,000 pre-school age children and 1.6 million school-age children in need of treatment each year [17,18]. In Benin, 72% of households report limited access to sanitation and 40% of households live below the poverty line [15,16]. The Gender Inequality Index (GII), a measure of gender inequities that range from 0 to 1, ranks Benin 146^th^ out of 180 countries with a GII score of 0.611 [19]. For example, in Benin 18.2% of women receive secondary education, as compared to 32.7% of men, and men are twice as likely to be literate. [20,21].

### Sampling

Four of the twenty DeWorm3 intervention clusters in Benin were randomly selected for inclusion in this study. Within each selected cluster, twenty adult community members aged 18 and over (10 men and 10 women) were randomly selected from an exhaustive population census completed within the DeWorm3 project (Table 1). In each cluster, FGDs were conducted separately for adult men (4 FGDs conducted) and adult women (4 FGDs conducted). Selected individuals were called and invited to participate and, in the event of refusals, subsequent individuals on the sampling list were contacted until the targeted sample size was reached. No more than one individual per household was sampled. Participants were provided with compensation to cover transportation and their time for attending.

**Table 1:** Overview of FGDs sampling.

Stakeholder	Sampling Frame	N
Men	Males 18 years of age or older randomly selected from four DeWorm3 intervention clusters	4 FGDs; total 40 participants
Women	Females 18 years of age or older randomly selected from four DeWorm3 intervention clusters	4 FGDs; total 38 participants
Community Drug Distributors	Male and female trained CDDs with experience delivering MDA were randomly selected in the four selected community clusters	2 FGDs; total 17 participants (4 women, 13 men)

Two additional FGDs were conducted with CDDs. Participating CDDs were identified and randomly selected for inclusion based on the criteria that they must have been formally trained by the MOH and have participated in at least one previous MDA campaign prior to their engagement with the DeWorm3 Project. Over 20% of CDDs (4/17) participating in the FGDs were female, which is slightly higher than the 14% of CDDs in Comé, Benin who are female (24/171).

### Focus group discussion procedures

The DeWorm3 project’s primary qualitative data collection, from which this substudy stems, is informed by the Consolidated Framework for Implementation Research (CFIR). The CFIR is a metatheoretical framework comprised of 39 constructs across 5 domains that influence implementation and implementation effectiveness [22]. Select CFIR constructs used to inform the question guides for the parent study. The same question guide was used for male and female FGD participants, and a similar but adapted question guide was used for CDDs. The question guides did not ask participants to explicitly reflect on gender differences, rather the purpose of this study was to compare and contrast responses by gender.

The FGDs were held in April and June 2018, prior to the first round of DeWorm3 MDA. Interviewers were trained Masters-level Beninese social scientists, who led question guide adaptation at baseline and worked with senior social scientists to iteratively review audio recordings and refine their FGD facilitation skills over time. FGDs were conducted at various locations in each community, including private classrooms, meeting rooms, and the chief’s residence. FGDs were recorded and additional notes were taken by a second facilitator. After the FGDs, both the audio file and typed notes were uploaded into Dropbox and a secure hard drive [23]. Interviews were conducted in French or a local language (Mina and Watchi) and first transcribed before being translated into English by trained professionals in Benin. Both transcriptions and translations underwent quality assurance reviews to ensure accuracy throughout this process, any discrepancies were referred back to the original transcriber or translator for revision.

### Analysis

The Women’s Empowerment Framework (WEF) was used to inform our analysis [24]. The WEF is based on the concept that equal participation in health and development programs empowers women [24]. This framework incorporates five constructs that build upon each other to inform a nuanced understanding of gender empowerment: welfare, access, conscientization, mobilization, and control.

**Fig 1:**
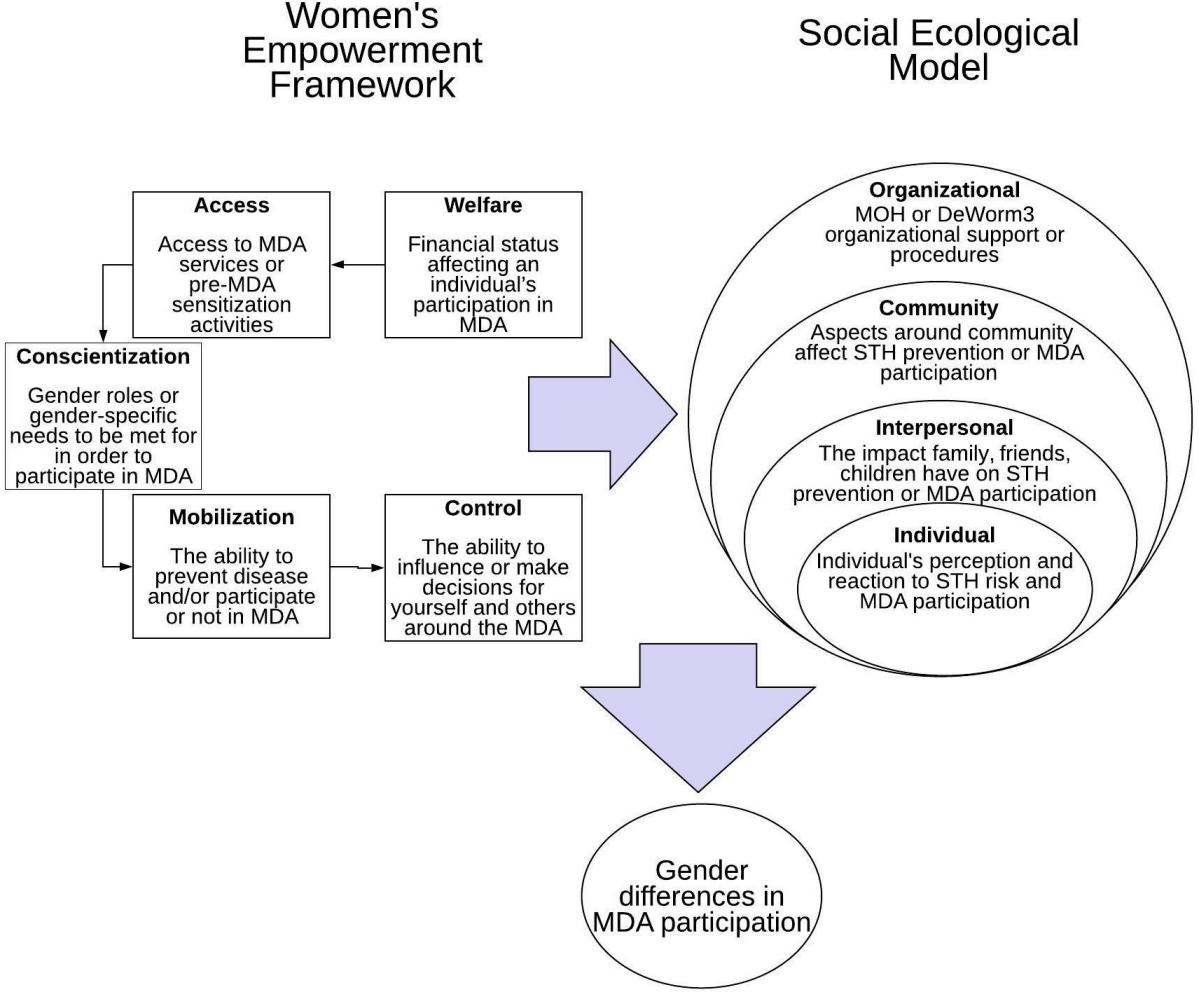
Conceptual framework combining Women’s Empowerment Framework (WEF) with the Social Ecological Model (SEM).

Definitions of each WEF construct, tailored to MDA for STH, are presented in Fig 1. “Welfare” involves how an individual’s economic or financial status affects his or her health and the ability to obtain healthcare or participate in MDA [24]. “Access” involves the distribution of resources and access to MDA information or pre-MDA sensitization activities. “Conscientization” refers to the ability to understand differences between sex and gender roles and recognize any gender-specific needs related to MDA. “Mobilization” focuses on one’s ability to put into action methods to prevent disease and to participate in MDA. Lastly, “Control” relates to one’s ability to make decisions about disease prevention and MDA participation. As conceptualized in the WEF, “Welfare” is the most basic form of gender equity, while “Control” is the ultimate form of equity. Within the context of MDA treatment coverage, each construct of the WEF was differentiated in the coding process as negative (construct is perceived as a barrier to participation) and positive (construct is perceived as a facilitator to participation). In order to conceptualize potential contextual influences of gender-specific barriers and facilitators to treatment, we also coded FGDs content that pertained to levels of the social-ecological model (SEM) [25]. The SEM elucidates the broader context of the planned MDA intervention, from population or global influences down to personal influences [25]. Each of the levels (organizational, community, interpersonal, and individual) impacts the next, so health interventions are believed to be more successful when interventions address the barriers and facilitators present at all levels. This two-component conceptual framework (i.e., WEF and SEM) was used to conduct a gender-based analysis (GBA) and identify gender-specific similarities and differences in facilitators and barriers to MDA treatment coverage, and to develop an understanding of how these barriers could influence STH transmission interruption programs.

ATLAS.ti 8 qualitative software was used to store and organize the transcripts [26]. The codebook was prepared *a priori*, based upon the WEF and SEM frameworks, and a mix of deductive and inductive coding was used. The codebook was updated iteratively by the coders to provide clarity and inclusion of the inductive themes that were identified during the coding process. A group of six coders based in Seattle, Washington, United States and Cotonou, Benin engaged in data analysis. Two coders and a tie-breaking coder were assigned to each transcript using an Excel-based tracking tool. All coders were trained on data analysis using standardized analysis plans and standard operating procedures. All transcripts were double coded and the coders met weekly to discuss any variabilities in coding and to resolve differences. An additional third party reviewed discrepancies in codes and served as a tie-breaker. Saturation in coding was determined when no new emerging themes were identified and no new codes were added during analysis, across coders.

Once all transcripts were coded, case memos were created separately by two coders for each group (e.g., men, women, CDDs) to document salient themes. Memos addressed each construct in the conceptual framework and included (1) a summary of main findings within each group, including breakdowns by SEM level, (2) a short rationale or justification for the summary, and (3) supportive coded statements from the relevant data.

## Results

This study found many similarities in the perceptions of men and women regarding community-based MDA for STH, as well as several notable distinctions. Both genders expressed concern that deworming drugs were not affordable outside of campaigns and that the community would benefit from packaging MDA along with other health services, such as water, sanitation, and hygiene (WASH). Women perceived a gap between the organizations involved in MDA delivery and communities they served, which left many feeling that community-wide distribution programs were more disorganized than beneficial leading women to express desire for increased involvement in community-based public health programs to improve program design and delivery. Alternatively, men perceived themselves to be at lower risk for STH compared to women and children and did not express a demand for the MDA. Overall, CDDs only identified barriers presented by both genders. These themes are further elaborated, with representative quotes, in the sections below.

### Both men and women seek yet struggle to afford deworming drugs outside of MDA campaigns

Across all FGDs, participants emphasized that they were aware that STH is present in their community, but they may not be able to consistently obtain deworming medications. Many of the adult FGDs participants, who were not targeted by the school-based distribution programs, reported seeking and purchasing deworming medicines for their families independent of government sponsored MDA programs. Participants of both genders noted that when they were not able to afford deworming drugs, they resorted to brewing traditional herbal teas (made from dried papaya seeds) that are widely believed to treat STH in Benin.

“*Even if you go to the hospital*, *they will prescribe the medicine you have to buy*. *Nothing is for free*. *They will just prescribe some drugs to buy*. *If you do not have money*, *you become anxious and you have to resort to herbal teas*.*”–Woman 4*, *Cluster 2*

Women whose primary income was generated by food services reported that their financial welfare was negatively affected by poor access to affordable deworming medicines. One participant said that some women fear that they will lose their food vending licenses if they are suspected of having infectious health problems, including helminth infections.

“*If it is this disease that has been diagnosed… they will then forbid you to prepare*. *On your paper*, *they will write with red pen and will not give it back to you*. *You will no longer cook*. *It is the law that forbids it*. *They will write a report*, *add your photo and send them to the town hall with as instructions that you are no longer allowed to cook and you should be arrested if you are found doing so*. . . . *Your trade will be at a loss*.*”–Woman*, *Cluster 1*

Unlike women, who focused on the health and individual or interpersonal consequences of not receiving treatment, men were more likely to discuss financial welfare in a general context by discussing strategies for how community members can privately procure deworming medications. For example, some men expressed that treatment of adults in the community is erratic due to the cost and challenges of purchasing deworming drugs from private pharmacies.

The CDDs also discussed the financial burden associated with obtaining treatment for individuals who were not targeted by previous distribution programs and highlighted that the burden is shared amongst people of both genders. The CDDs commented that community-wide MDA would be widely beneficial to those who typically could not afford treatment or to afford to send their children to school where they would be treated by the school-based distribution programs.

“*It also allows parents to moderate their budget a bit*. *Otherwise*, *without this favor*, *I mean*, *if every time we had to go to the pharmacy to buy drugs*, *and mainly*, *here*, *we wait for the disease to reach a certain harmful level before we treat it*, *so it can still worsen children health*. *Now that there is this opportunity*, *that we enjoy*, *that include[s] free distribution of drugs*, *it lessens parents’ job*.*”–Man 2*, *CDD*

### Both genders feel the MDA should be packaged with WASH services

The CDD participants reported that a lack of WASH resources might negatively affect community-based MDA coverage. For example, their prior experiences indicated that community members may not want to swallow the drugs with water perceived as unsafe as they had often asked that the distributors provide clean water for them. Without provision of safe water, the CDDs feared that individuals would refuse to participate in community-wide MDA.

“*We lack well[s]*, *even SONEB [National Water Company of Benin] does not have its network here in the villages*, *that's why villagers will ask us [for] drinking water to take drugs*. *We have problems in the community”–Man 6*, *CDD*

Feedback from male and female community members supported CDD statements; several participants were concerned that MDA is just a short-term solution if sources of infection such as unsafe water and open defecation persist in their communities. In this sense, both genders had a negative perception that community-wide MDA programs were not comprehensively meeting their needs.

“*If it's a medicine you're going to put inside so that we can drink that water without fear*, *please*, *do it*. *… If we have to treat the water of the well so we can use it until the fountain is made*, *do it for us*. *Latrines are important too*. *To [defecate] or urinate now*, *you can only do that on the ground*. *When it rains*, *it flows with runoff water…it goes directly into the well and this is the water that you will still fetch for drinking; so try to help us*.*”–Woman 2*, *Cluster 1*

Community members of both genders agreed that they felt knowledgeable regarding options for individual-level STH prevention (ex. handwashing, washing of food, avoidance of open defecation). Both genders noted that they felt more comfortable with individual-level prevention methods than community-level treatment. They discussed at length the methods they used to prevent STH in themselves and their families and wanted more emphasis placed on addressing these prevention activities by the DeWorm3 Project implementation. Of note, both genders felt that personal preventative measures such as washing and preparing food were a women’s responsibility.

“*But if there is a lack of hygiene for oneself*, *for children or grandchildren*, *it is a problem; you cannot be well*. *It is necessary to keep the drinking water*, *the water for cooking and water for the laundry and the dishes in good conditions*. *These are the means of prevention of the diseases*.*”–Woman 7*, *Cluster 2*

### Women feel community-wide programs in the past have been disorganized

Women reported that prior community-wide distributions have been disorganized, and they do not feel motivated to participate in future campaigns. Some women stated that they have tried to participate in community-based public health campaigns in the past that utilized central distribution points, but the campaigns were not well organized, with people waited for long periods of time, sometimes without ever receiving the required drugs. In contrast, only one of the male participants discussed the timing and location of distributions as being a community-level barrier to treatment. Women felt that waiting to receive MDA medicines in this manner was not a good use of their time and they noted that, instead, door-to-door MDA removed participation burdens placed on women in particular. Women said that they often serve as “Heads of Distributions” for their families, thereby influencing the distribution of the drug to the entirety of their family and individual-level compliance within the family. In some FGDs, women discussed the role of the mother specifically in monitoring and maintaining her children and family’s health and reported that central distribution points do not empower them to make treatment decisions for their whole family.

“*If we have to meet at the delegate’s* … *for example*, *when we distributed mosquito nets*, *and I already talked about it* … *We could not take this because there was too much mess*. *We stayed under the hot sun*. *The sun was burning*. *If you sit until nightfall*, *you'll leave*.*”–Woman 5*, *Cluster 1*

While men offered minimal feedback regarding community sensitization activities, women provided thorough responses regarding how communities can be more fully engaged in community-wide MDA. For example, several said community sensitization for prior community-based programs have been insufficient and frustrating. Many stated concerns that their geographic location in the village in relation to the chief’s palace or other major convening points utilized by town criers would impede their ability to receive pertinent information about the programs. They highlighted that if they did not receive sufficient information about the time and place of imminent MDA programs, they would not be able to ensure that their families are able to participate.

“*Maybe the person may have an impediment on the day of the drug distribution*, *or maybe she is not informed because if she was absent the day the town crier passed the information; or if it came after the closing time of the distribution*. *For example*, *when distributing mosquito nets*, *I was not served simply because I was not home when the agents were passing through*.*”–Woman 3*, *Cluster 3*

### Women desire increased engagement in community-wide MDA programs

A common theme throughout all of the women’s FGDs was discontent with the lack of control and engagement of female community members in community-wide MDA programs. The discontent appeared to stem from negative experiences with school-based distribution. The mothers voiced concern that they did not know when their children were receiving drugs, what drugs they were being given, and if their child actually swallowed the drug or not. The mothers felt as though school-based distribution compromised their control over the health of their family.

“*Just that none of my children have ever taken the drug at school and I do not know what medicine it is*. *So*, *I have forbidden my children to take it”–Woman 6*, *Cluster 1*

These concerns were compounded by stories of children who suffered side effects after taking the drugs and needed to be taken to the health center. Several mothers and fathers stated that they exercised their control by keeping their children home from school during MDA delivery days or forbidding their children from swallowing drugs provided. A perceived lack of involvement, by women, and sensitization, by men, were primary rationale for parents to reject previous rounds of school-based MDA and participants clearly stated that this should be addressed in order for the community-based MDA to succeed.

When discussing school-based drug distribution, both genders expressed dissatisfaction with their lack of involvement. However, women were more focused on interpersonal relationships with their children and their own decision-making capacity about whether their child would participate, while men focused on organizational dynamics regarding how schools deliver MDA without informing parents first. In multiple FGDs, women stated that they should be present during MDA distributions to ensure that their child has eaten and taken the correct dose of the drug. They recounted how their lack of involvement in the school-based distribution led to some children spitting out tablets, whereas if the mother was involved, she could monitor that the entire dose was taken. While men voiced their frustrations around the current school-based program, they did not specifically state specific mechanisms through which they want to be more involved in community-wide MDA programs.

“*I see that it [school-based distribution] is not a good thing because everyone has their way of educating their child*. *If they had come to our homes to give the drugs to the children*, *the parents would have known … the tablets [are] swallowed by the children”–Woman 1*, *Cluster 2*

Most women believed that, if given the opportunity, they would be able to oversee dosing of their families’ deworming medications and obtain medication for family members unavailable at the time of the distribution, thus ensuring that every person would be treated in the household. They reported the desire to better control their children’s healthcare and emphasized that they would be more likely to consent to their child’s participation in MDA for STH if treatment took place in the home through a door-to-door approach as opposed to delivery in schools.

“*In principle*, *if we had to respect the texts*, *before giving medicine to a child*, *we should ask him to go and call his mother*. *The mothers of the children should be present during the distribution*. *Every mum knows how she has educated her child*. *But how can a child respect the dosage of tablets you put into his hands*? *He might take two tablets instead of just one*. *That can cause problems; it is not logical*.*”–Woman 9*, *Cluster 2*

Women reported that they could positively influence mobilization for community-based programs. Several women suggested that treatment coverage is lower when women’s perspectives are not considered prior to MDA distribution. These women believed that engaging women in MDA planning and administration would be an extension of their current roles managing their family’s health.

“*The disadvantage that we may have is*, *for example*, *the non-involvement of mothers during the distribution of drugs*. *They are the ones at home*, *the drivers in the homes*. *Mothers should be given medications based on the number of people in the household*. *She is the one who will manage the medication*.*”–Woman 2*, *Cluster 3*

Many women also noted that they could assert influence on treatment participation of community-wide MDA programs via their social networks, including their personal and professional networks.

“*If possible*, *we will also give information to other women about the benefits of the treatment*. *We can help you pass the information to our girlfriends*. *I have my shop at a crossroads*. *I will speak with my male and female clients and even with children”–Woman 6*, *Cluster 2*

In contrast to women’s statements about previous lack of involvement deterring them from accepting past school-based MDA, many of the men’s statements involve a lack of trust in the health authorities and what drugs they claim to be providing. Some men believe stool samples should be collected prior to drug distribution in order to determine that the correct drug is being given. Others stated that they will “categorically refuse” to accept drugs.

“*They distribute spoiled drugs*. *The distributed drugs are normally supposed to heal us but it is the opposite*. *It is rather to kill us*. *That’s why I refuse all these kinds of drugs and vaccinations*. *What my eyes have seen is too much*.*” Man 6*, *Cluster 1*

### Men perceive themselves to be at low risk for STH

In the FGDs, men more readily discussed procuring deworming drugs for their children, rather than for themselves. Some men even stated that while they buy deworming drugs, they cannot recall the last time they treated themselves with the drugs. Male participants often emphasized that children are primarily at risk of STH and are the ones who require timely treatment.

“*But*, *when it comes to intestinal worms*, *it's mostly children who suffer from it*, *and even me here*, *I cannot even tell you the last time I took de-wormers again*. *It’s been more than 5 years now*, *I took deworming drugs again … worms only attack those age groups*,*”–Man*, *Cluster 3*

Some men suggested that women may also be at greater risk of STH infection; there are prevailing beliefs that eating sweet foods such as mangoes leads to STH infection or worm reproduction and men stated that women are more likely to eat these foods. These misconceptions about risk and transmission may impact men’s willingness to participate in the MDA if they do not consider themselves vulnerable to infection. Compared to women, male FGD participants were more likely to be distrustful of MDA or declare that they were unsure if they would participate in the DeWorm3 program’s community-wide MDA campaigns. However, they did not provide insight on a preferable approach to MDA design.

“*There is no benefit in this treatment*. *I took the medicine and it did not help me*. *We must tell one another the truth*. *Even if you give me the drugs here now*, *I’ll throw them on the way home*. *I still have bad memories*.*”–Man 6*, *Cluster 1*

Nearly all the male FGDs stated how STH infections in a household reflect poor hygiene and eating habits on the part of the mother, as they are the primary caregiver to children. These men stated that a mother’s lack of hygiene increases their child’s risk for STH infection.

“*So*, *when the mothers of children lack a little hygiene and that the children walk*, *are in contact with the ground and underpants*, *all that causes the disease of the worms*.*”–Man*, *Cluster 3*

### Community drug distributers were poorly equipped to identify gendered barriers

The school-based STH program was a major concern for both men and women within these communities and the primary rationale for women expressing a need for increased involvement in MDA sensitization and distribution. However, CDDs did not mention these community frustrations around school-based distribution. Most CDDs expressed concerns regarding potential refusals associated with community member knowledge of STH or the influence of religious institutions and leaders on community member refusals. However, these concerns were not discussed by either male or female community members.

CDDs believed that they may be able to bridge the gap between MDA programs and community members who may be reluctant to engage in MDA. CDDs are members of the communities they serve and one CDD articulated that the selection of distributors will influence community trust and, ultimately, treatment coverage.

“*For example*, *if…it’s foreigners that you have sent to these villages*, *we can have difficulties but at home*, *there are not too many difficulties compared to what is prohibited*. *We already know each other*, *even the villagers know us*, *we know our village*.*”–Man 2*, *CDD*

More female CDDs may be needed in order to address issues of low treatment coverage and deliver MDA programs that meet the needs of all genders. Only one of the female CDDs discussed a gender-specific facilitator identified during the CDD FGDs; she believed there is untapped potential for women and mothers in particular to contribute to improved MDA treatment coverage by conducting sensitization within their immediate households and social circles. By not addressing gender-specific facilitators and barriers, CDDs will not be able to provide the support and guidance all community members need to maximize treatment coverage and compliance.

“*It is in our communities*, *the time women stay at home*, *we must know these hours… those who are around*, *we will work with them*, *if it is the in night that others stay at home*, *so we will… continue with them”–Woman 2*, *CDD*.

### Summary of themes in the context of the WEF and SEM

The contextualization of emerging themes within the WEF and SEM is outlined in Table 2 and Table 3, presenting facilitators and barriers to MDA access and participation, respectively. When discussing the construct of welfare, women indicated more individual-level drivers for participating in MDA included access to free treatment that would alleviate financial burdens associated with pharmacy costs. This facilitator was activated at the community-level for men where discussions centered around facility or pharmacy-based interactions and was not discussed as an individual-level concern. For women, facilitators to treatment were primarily at the interpersonal level in that community-wide door-to-door MDA would provide women more control over the health of their children and family members. There were no facilitators that emerged for men at either the individual or interpersonal levels. At the community level, women prefer the flexibility that door-to-door MDA provides them, since it would not require them to wait at a central location all day; while men prefer any MDA strategy that reduces their need to purchase drugs at the health centers. At the organizational level, distrust of standard school-based distribution model acts as a potential facilitator of engagement in community-wide MDA for both men and women. More specifically, community-based distribution might allow women to exercise greater control over their family’s health and wellbeing. Thus, facilitators were clustered around WEF concepts of welfare and control and in the SEM at interpersonal and community levels. Women identified many more facilitators to MDA participation than men.

**Table 2:** Summary of facilitators towards participating in the MDA broken down by gender.

	Welfare	Access	Conscientization	Mobilization	Control
Individual	W: Inability to purchase drugs negatively affects individual welfare; community-wide, MDA will alleviate this burden				
Interpersonal			W: As family caregivers and heads of household, women can ensure high treatment compliance		W: Want to be empowered to make decisions about their children’s health
Community	M: Drugs are expensive at facilities, which increases demand for community-wide MDA			W: Door-to-door MDA allows greater flexibility in meeting caregiving and income generating responsibilities	
Organizational					M: Distrust of the current school-based system causes men to favor community-based MDA platforms

W: Women, M: Men

**Table 3:** Summary of barriers towards participating in the MDA broken down by gender.

	Welfare	Access	Conscientization	Mobilization	Control
Individual			W: Feel personally unengaged in MDA design and delivery M: Do not perceive themselves to be at risk, and thus do not prioritize self-treatment	Both: Feel comfortable with personal prevention strategies (ex. handwashing), causing some doubt regarding the necessity of treating adults	
Interpersonal			M: Believe women are the major source of infection for children, and men are at low risk and thus may not require treatment		
Community		Both: Lack of WASH resources to prevent STH (not bundled into MDA) reduces perceived quality of MDA			
Organizational		Both: Lack of MDA sensitization reduces demand		W: Perception of MDA has been negatively colored by disorganized distributions in the past	W: Feel unempowered by community-based public health programs M: Want to be more informed by the organization prior to MDA

W: Women, M: Men

Barriers present at the individual level were that both genders felt comfortable with their own deworming prevention routines and may not see an urgent need for deworming of adults. Additionally, women felt unengaged and that their individual needs were not being met by MDA design and delivery while men did not see themselves as a risk and therefore do not prioritize participating in MDA. Only men discussed interpersonal barriers to participating in MDA; they suggested that women are a primary source of STH infection for children and that treatment is not urgent for men because they are at lower risk of infection, as a result. The only barrier at the community-level was that both men and women perceived that WASH services need to be packaged alongside the MDA, in order to increase the legitimacy and effectiveness of a comprehensive STH package and improve participation. Finally, at the organizational level, barriers to MDA participation included lack of information by both genders, lack involvement of women within the program and distrust of deworming drugs due to negative past experiences with MDA. Barriers were more skewed towards the higher end of the WEF, with the more prominent gender-specific barriers characterized under conscientization and control concepts.

## Discussion

This study attempts to capture and summarize the perspectives of women and men in communities across Comé, Benin as well as the perspectives of CDDs who are working to reach them during community-wide MDA. These perspectives are needed to help identify the gender norms influencing MDA coverage and opportunities for overcoming gender-associated barriers to treatment. In this study, we found that while both men and women were open to a new community-wide approach to deworming, they still had some reservations about implementation.

Both men and women indicated that standard-of-care school-based deworming programs were problematic, however they cited different supportive rationale. Women perceived school-based distribution programs as disempowering of mothers, as they could not observe their children’s treatment and ensure it was taken. The current school-based distribution protocol includes sensitization of parents about the MDA, but men remarked that these efforts are insufficient as they were still not receiving necessary information prior to distribution. Women also welcomed the door-to-door approach to STH MDA as it allowed them to exert more control over how and when deworming drugs were given to their children. This finding is supported by a study in Kenya which found that parents were not sufficiently informed about MDA and that some teachers were not properly trained prior to distribution, causing the community to be dissatisfied with the design of extant school-based deworming programs [27]. A door-to-door MDA strategy may assuage some of these parental concerns. Additionally, based on parental feedback provided in this study, door-to-door distribution may also improve pediatric treatment coverage by improving program acceptability. This may have important implications for improving both equity and progress towards control and elimination targets outlined in the London Declaration [7].

Additionally, women felt they were underrepresented in the planning process and during implementation. These findings are consistent with evidence from other settings and health foci. Several studies have identified associations between measures of female empowerment (defined as an ability to influence one’s welfare and make household decisions, among other self-reported factors) and improved health outcomes [28,29]. A desire for more control was the predominant sentiment of female FGDs and may reflect social norms and dynamical challenges above and beyond delivery of MDA programs. Thus, including women in the design and delivery of community-based STH MDA might be an opportunity to improve gender equity and contribute to women’s empowerment more broadly.

Men did not perceive themselves to be at high risk of STH infection, and thus demand for unprogrammed deworming medicines or access to community-based STH MDA was perceived as lower amongst men as compared to women. This is consistent with other studies in which men identified themselves as being less susceptible to diseases such as malaria, in comparison to women [30]. Furthermore, many of the men perceived women to be responsible for family hygiene and therefore to blame if they or their children become infected. Previous studies compare how typical gender roles, such as preparing food, may perpetuate perceived differences in disease risk between men and women [30, 31]. The different responses from men and women while discussing mothers’ role in STH prevention and treatment highlighted clear inequitable gender roles that may need to be addressed to achieve high treatment coverage.

Our finding that women may play a critical role in household MDA treatment coverage confirms reports from other studies. For example, Krentel et al. examined the dynamics surrounding couples’ decision-making to participate in lymphatic filariasis (LF) MDA program in Indonesia [10]. They reported that women, especially mothers, play a critical role in decisions regarding household MDA participation and are more active than men in ensuring that their families are treated during MDA [10]. We found that women tended to be the ones responsible for representing their households during community-based public health campaigns, and that men were less likely to fulfill this role. Women were strongly in favor of door-to-door distribution of deworming drugs, as it allowed them to utilize their time efficiently while still receiving the distribution and engaging in the treatment of their entire family. While the door-to-door MDA framework may be preferable to women in this study in Benin, a study from Uganda found that men face more barriers in receiving information and distributed medications via door-to-door campaigns than women [11]. In our study it was not evident that door-to-door MDA would influence treatment seeking behaviors by adult males.”

Intersecting axes of inequity between factors such as geographic location within a community, socioeconomic status, education, and religion may also exacerbate gendered inequities to MDA access. For example, female FGD participants expressed concern that living on the periphery of a village would prevent them from receiving MDA sensitization messaging. Additionally, poverty and income generating opportunities were major concerns for female participants. These challenges to equity and healthcare interact with gender in complex ways, highlighting the importance of addressing intersectional barriers to access as a whole, rather than in isolation [12]. While differences in gender were highlighted in the results, some subjects that were prominent in the focus groups varied slightly between clusters. For example, the clusters that were more rural spent more time discussing the need to improve WASH resources.

CDDs have a critical influence on MDA treatment coverage via their engagement with the community and knowledge of community norms and preferences [10,12,32]. The CDDs in this study discussed potential barriers to effective MDA delivery that were common to both genders, such as concerns regarding comprehensiveness of MDA programs and the financial burden of purchasing deworming treatment. Only once did a (female) CDD capture a gender-specific facilitator regarding opportunities to engage women to increase MDA treatment coverage. However, the majority of the participating CDDs in this study were male, which may have influenced their perceptions of facilitators and barriers faced by both men and women in the community. This is likely due to the fact that FGD guides were not specifically designed to delve into gender-specific content. Several studies have similarly identified that male and female CDDs approach MDA delivery activities such as community sensitization differently and thus an awareness of the preferences and needs of each gender may be an important addition to CDD training activities [12,33]. A more gender-equitable CDD workforce may have a more balanced representation of the priorities and concerns of all genders, with positive effects on MDA treatment coverage. Because CDDs are volunteers in Benin, it may be challenging to build a gender-balanced CDD network and, in the meantime, it is important that a comprehensive training is provided to CDDs that accounts for gender-specific barriers to treatment.

Women often emphasized opportunities to contribute to or increase ownership of community-wide MDA activities; within the WEF framework, equity in “control” is the final step in reaching women’s empowerment and promoting women’s involvement in these programs may be an avenue for increasing gender parity more generally throughout the community.

Compliance has been used throughout this paper to convey the action of taking deworming drugs. However, there is some discussion around using the word “compliance” over of other terms such as “adherence” that imply a more active decision-making process or shared agreement in receiving medications between the distributor and individual. However, in order to maintain consistency with current NTD literature, compliance is the most accurate term for use within this context [33,34]. This study had several limitations. The FGDs took place prior to the first round of DeWorm3 community-wide MDA for STH (i.e. formative research), and barriers and facilitators to community-wide MDA were based on participant’s previous experiences with other programs (i.e. school-based STH programs or community-wide MDA for other NTDs). Findings from this study will be triangulated with qualitative data collected following community-wide MDA in the DeWorm3 Project, which will strengthen study findings. Additionally, the topic guides used by facilitators for the FGDs were not originally designed to delve specifically into differences in gender perceptions around community-wide programs. These results may not necessarily be transferable across communities in Benin or other NTD-endemic setting, however the exercise of comparing experiences and insights across men and women is generalizable, and this study suggests that it is a valuable activity that can yield useful information to guide program adaptation and responsiveness to the gender norms of a given community. Random sampling was employed for the FGDs rather than purposive selection based on demographics or experience in order to attain a representative sample of community members and CDDs in this large catchment area In this setting, random sampling was possible because the DeWorm3 Project had an exhaustive list of community members and engaged CDDs. Additionally, the qualitative data were collected in French or local languages (Mina and Watchi) and thus some dialect-specific nuances may have been lost in translation.

This study points to specific recommendations for creating more gender equitable community-wide MDA programs that strategically incorporate opportunities to empower women. For example, door-to-door MDA campaigns may be more responsive to women’s needs and priorities by providing women control over their family’s health and improved access to the distribution, while not monopolizing their time. Findings from this study also demonstrate that perceived barriers and facilitators of community-wide MDA for STH may differ between genders, potentially influencing participation in community-wide MDA for STH, or community-based public health programs more generally. These findings can inform future community-wide MDA campaigns to increase access and coverage for both men and women living in STH-endemic areas. Women often emphasized opportunities to contribute to or increase ownership of community-wide MDA activities; promoting women’s involvement in community-wide MDA programs may be an avenue for increasing gender parity more generally throughout the community.
